# Development and validation of a model for early prediction of severe/critical COVID-19 in elderly patients

**DOI:** 10.7717/peerj.21417

**Published:** 2026-07-09

**Authors:** Jiaxuan Li, Ruining Li, Chang Hong, Richeng Mao, Lushan Xiao, Ziyong Zhang, Min Ding, Xuejing Zou, Li Liu

**Affiliations:** 1 Department of Health Management, Nanfang Hospital, Southern Medical University, Guangzhou, Guangdong, China; 2 State Key Laboratory of Organ Failure Research, Department of Infectious Diseases, Nanfang Hospital, Southern Medical University, Guangzhou, Guangdong, China; 3 Department of Infectious Diseases, Shanghai Key Laboratory of Infectious Diseases and Biosafety Emergency Response, National Medical Center for Infectious Diseases, Huashan Hospital, Fudan University, Shanghai, Shanghai, China

**Keywords:** COVID-19, Risk stratification, Logistic regression, Predictive model, Personalized medicine

## Abstract

**Background:**

The mortality rate of severe/critical coronavirus disease (COVID-19) is high in the elderly, and early prediction of its prognosis can facilitate timely treatment and reduce mortality. This study aims to identify early predictors of severe COVID-19 in elderly and construct a validated risk prediction model.

**Methods:**

This retrospective study included 722 elderly COVID-19 patients (those aged ≥60) who attended Nanfang Hospital of Southern Medical University between July 2022 and November 2023. They were categorized as mild/moderate or severe/critical according to the extent of their condition during hospitalization. Predictive models were constructed using logistic regression analysis and visualized using nomograms. Receiver operating characteristic (ROC) curves were used to assess the model’s accuracy and predictive value. An external validation cohort containing 1,249 elderly COVID-19 patients who were admitted to Huashan Hospital of Fudan University between March and May 2022 was also collected.

**Results:**

In multivariable logistic regression analysis, respiratory rate, comorbid diabetes, C-reactive protein (CRP), lymphocyte percentage, and D-dimer were independently associated with severe and critical COVID-19. Based on these findings, the final severity prediction model was constructed using three laboratory markers: CRP, lymphocyte percentage, and D-dimer. This model achieved an area under the curve (AUC) of 0.753 (0.713–0.794). For mortality prediction, CRP and D-dimer emerged as the significant independent predictors; the model showed an AUC of 0.722 (0.653–0.791) in the internal validation cohort and 0.877 (0.833–0.921) in the external validation cohort.

**Conclusions:**

The predictive model incorporating features selected *via* logistic regression accurately predicts prognosis of severe COVID-19 in elderly, facilitating the implementation of early clinical interventions.

## Introduction

Coronavirus disease 2019 (COVID-19), caused by severe acute respiratory syndrome coronavirus 2 (SARS-CoV-2), has transitioned to a phase of post-pandemic normalized management globally. While COVID-19 is no longer a national public health emergency, the continuous emergence of new SARS-CoV-2 variants means COVID-19 still poses a significant health threat to vulnerable populations, particularly the elderly. By the end of 2023, global infections had exceeded 700 million with over 7 million deaths (Chung et al., 2024). Recent surveillance data highlight this persistent burden: an August 2025 report from the Chinese Center for Disease Control and Prevention (China CDC) documented 164,625 new COVID-19 cases, confirming it as the morbidity leader among notifiable infectious diseases (Chinese Center For Disease Control and Prevention, 2026a). In China, adults aged 60 and older exhibited increased disease severity and mortality rates (Lian et al., 2020). In a study conducted across 16 countries and regions, it was found that individuals aged 65 and above had significantly higher COVID-19 mortality rates compared to younger populations (Yanez et al., 2020). According to the U.S. Centers for Disease Control and Prevention (CDC), elderly individuals face the highest risk of severe COVID-19 and disproportionately represent 31% of cases, 45% of hospitalizations, 53% of intensive care unit (ICU) admissions, and 80% of deaths, the mortality rate in this group is 97 times higher than that of adults aged 18–29 (Centers for Disease Control and Prevention, 2026; Bialek et al., 2020).

Compared to younger patients, elderly patients demonstrated higher lung severity index scores, multilobar involvement on CT scans, higher incidence of acute respiratory distress syndrome (ARDS), elevated risks of cardiovascular and cerebrovascular complications, increased need for ICU admission and mechanical ventilation, and potentially shorter median time from symptom onset to death (Liu et al., 2020; Smorenberg et al., 2021; Wang, Tang & Wei, 2020). These patients often present with atypical symptoms such as fatigue, anorexia, and delirium rather than classic respiratory signs, complicating early diagnosis and timely intervention, exacerbating severe illness rates and placing ongoing pressure on healthcare systems (Limpawattana et al., 2016; Olde Rikkert et al., 2020). During the phase of routine management, early identification of elderly COVID-19 patients at risk of clinical deterioration is crucial for effective triage, appropriate allocation of medical resources, and the implementation of targeted interventions to reduce mortality.

The elevated vulnerability of the elderly is underpinned by distinct pathophysiological mechanisms. Advanced age is intrinsically linked to immunosenescence and a progressive decline in functional reserve across organ systems, which fundamentally impairs the ability to mount an effective immune response against the virus and increases the risk of systemic complications (Lewis et al., 2021). Furthermore, the presence of comorbidities (such as hypertension, diabetes, chronic obstructive pulmonary disease and cardiovascular conditions) imposes an additional load, compounding the physiological stress induced by SARS-CoV-2 infection and significantly elevating the likelihood of acute decompensation and severe outcomes (Sun et al., 2024). In addition, incomplete COVID-19 vaccination has been associated with a significantly increased risk of mortality (Pradhevi et al., 2024). The integration of these non-negotiable factors is therefore paramount for any prognostic model aiming to achieve accurate risk stratification in the elderly population.

However, the clinical implementation of existing prognostic models is significantly hindered by several limitations. A systematic review indicates that approximately 57.1% of previous COVID-19 prediction models were constructed based on single-center cohorts and relied solely on internal validation methods such as random data splitting (Appel et al., 2024). This practice is statistically inefficient and often results in overfitted models that fail to generalize to independent populations. Furthermore, many existing models are built using small sample sizes, resulting in unstable performance evaluation and unreliable risk prediction (Zhuang et al., 2023; Zeng et al., 2020). Most importantly, these models were largely derived from general adult populations with a much lower median age, failing to optimize for the unique physiological profiles and functional status of the elderly (Zahra et al., 2024).

The present study aims to address these critical research gaps by developing and externally validating a new nomogram specifically for elderly COVID-19 patients. Unlike prior efforts, we employ a large-scale, two-center dataset to ensure adequate sample size and implement rigorous validation processes across geographically distinct cohorts to guarantee generalizability. By integrating age, clinical, and laboratory indicators specific to the elderly population, we believe these nomogram models will provide clinicians with highly reliable, age-optimized routine monitoring tools, enabling the early detection of high-risk patients and promoting prompt clinical interventions.

## Materials and Methods

### Patients

This study was approved by Medical Ethics Committee of Nanfang Hospital of Southern Medical University (Approval No. NFEC-2026-166) and the Ethics Committee of Huashan Hospital of Fudan University (Approval No. 2025-856). The requirement for written informed consent was formally waived by the institutional review boards for this study. This study retrospectively analyzed the clinical data of 722 COVID-19 patients who were hospitalized in the Infectious Disease Ward and ICU Ward of Nanfang Hospital of Southern Medical University from July 2022 to November 2023, as well as 1,249 COVID-19 patients treated in the infection ward of Huashan Hospital affiliated to Fudan University between March and May 2022. Inclusion criteria: (1) age ≥ 60 years; (2) diagnosis of COVID-19. The exclusion criteria were as follows: (1) those with missing clinical data >20%; (2) those who were hospitalized for less than 24 h and discharged against medical advice.

### Diagnosis

Patient diagnosis and severity stratification are based on the Diagnostic Protocol for Novel Coronavirus Pneumonia (Trial 10th Edition) issued by the National Health Commission (Chinese Center For Disease Control and Prevention, 2026b). COVID-19 was diagnosed based on the following criteria: 1. Exhibits clinical manifestations consistent with COVID-19 infection; 2. Demonstrates one or more of the following pathogenetic or serological test results: (1) Positive COVID-19 nucleic acid test; (2) Positive COVID-19 antigen test; (3) Positive COVID-19 virus isolation and culture; (4) Recovery-phase COVID-19-specific IgG antibody levels showing a fourfold or greater increase from the acute phase.

The primary clinical outcomes of this study are severe/critical COVID-19 cases and in-hospital mortality. In-hospital mortality was defined as death from any cause occurring during hospitalization.

Mild/moderate patients meet the following criteria: (1) Primary presentation of upper respiratory tract infection, such as dry throat, sore throat, cough, fever; (2) Persistent high fever >3 days and/or cough, shortness of breath, but respiratory rate (RR) <30 breaths/min and oxygen saturation >93% while breathing room air at rest; (3) Imaging shows characteristic features of COVID-19 pneumonia. Severe/critical cases meet any of the following criteria in adults, with no alternative explanation unrelated to SARS-CoV-2 infection: (1) Dyspnea with RR ≥ 30 breaths/min; (2) SpO₂ ≤ 93% at rest while breathing room air; (3) Arterial oxygen partial pressure (PaO_2_)/inspired oxygen concentration (FiO_2_) ≤ 300 mmHg (1 mmHg = 0.133 kPa). In high-altitude areas (above 1,000 m), PaO_2_/FiO_2_ should be corrected using the following formula: PaO_2_/FiO_2_ × [760/atmospheric pressure (mmHg)]; (4) Progressive worsening of clinical symptoms with radiological evidence of >50% lesion progression within 24–48 h; (5) Development of respiratory failure requiring mechanical ventilation; (6) Development of shock; (7) Concurrent organ dysfunction requiring ICU monitoring and treatment.

### Data collection

We identified potential risk factors indicative of severe COVID-19 and indicators linked to COVID-19 mortality through prior research and clinical experience (Liang et al., 2020; Chow et al., 2020; Shang et al., 2020). These variables were considered potential predictors of poor prognosis in COVID-19. Clinical and laboratory information of participants was obtained from the electronic medical record system. The collected data comprised the following: (1) general data (sex, age, body temperature, pulse, respiratory rate, blood pressure at admission); (2) comorbidities; (3) laboratory markers (routine blood count, liver and renal function parameters, inflammatory indices, myocardial markers and coagulation parameters) in the first 24 h of hospital admission.

### Statistical analysis

Participants with variables having a missing rate exceeding 20% were excluded from the study cohort initially; for the remaining participants, k-nearest neighbor (KNN) imputation (with k = 5) was used to fill missing values of continuous variables, and the mode was adopted for imputation of missing categorical variables.

Normally distributed continuous variables were presented as mean ± standard deviation, while non-normally distributed continuous variables were expressed as median (interquartile range), and categorical variables were reported as percentages (%). Normally distributed continuous variables were analyzed using the Student t-test, whereas non-normally distributed variables were assessed using the Mann-Whitney U-test. Count data were expressed as frequencies or rates using the chi-square test. Statistical analyses were conducted using R software (version 4.4; The R Foundation for Statistical Computing), with significance defined as *p* < 0.05.

Univariate and multivariable logistic regression analyses were employed to evaluate the association between selected variables and disease severity. Variables yielding *p* < 0.05 in univariate analysis were deemed to possess potential predictive value and were selected for subsequent multivariable analysis using the “enter method”. Predictive models were constructed using logistic regression analyses and visualized using nomograms. The accuracy and predictive value of the model were assessed using receiver operating characteristic (ROC) curve analysis.

## Results

### Patient characteristics

**Figure 1:**
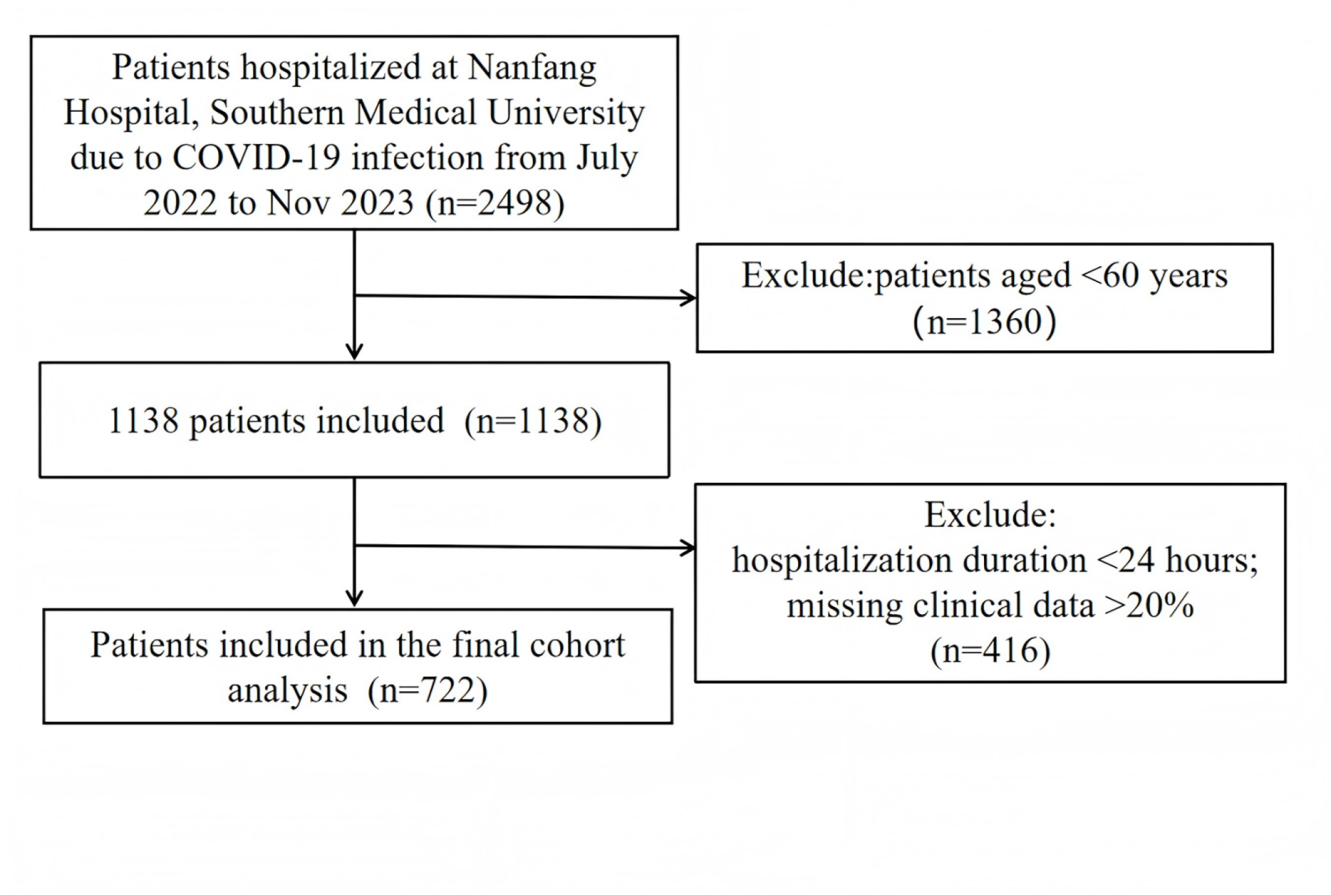
The flowchart of the study.

From July 2022 to November 2023, 2,498 COVID-19 patients were treated at Nanfang Hospital, among whom, 1,360 cases were excluded due to age <60 years, and 416 cases were excluded due to hospitalization duration <24 h or >20% missing clinical data (Fig. 1). A total of 722 COVID-19 patients from Nanfang Hospital were finally included, comprising 403 mild/moderate cases and 319 severe/critical cases, with 450 males and 272 females. The baseline information and biochemical examination results of all patients are presented in Table 1. The baseline data revealed statistically significant differences between the two groups in age, respiratory rate, pulse, body temperature, diastolic blood pressure, diabetes (*p* < 0.05).

**Table 1:** Clinical characteristics of elderly COVID-19 patients in Nanfang Hospital.

Clinical characteristics	Mild/moderate (*n* = 403)	Severe/critical (*n* = 319)	*p*
Sex			0.9781
Male	251 (62.3%)	199 (62.4%)	
Female	152 (37.7%)	120 (37.6%)	
Age, y	71 (65, 79)	75 (68, 83)	<0.0001
Respiration rate, breaths/min	19 (18, 20)	20 (18, 23)	<0.0001
Pulse, beats/min	85.5 (75, 96)	89 (78.25, 102)	0.0003
Body Temperature, °C	36.5 (36.3, 36.8)	36.6 (36.4, 37.0)	0.0306
Systolic Blood Pressure	129 (115, 142.3)	127.5 (112, 141.8)	0.2652
Diastolic Blood Pressure	75 (69, 83)	72 (65, 80.75)	0.0009
Hypertension			0.0658
Yes	215 (53.3%)	192 (60.2%)	
No	188 (46.7%)	127 (39.8%)	
Diabetes			0.0314
Yes	115 (28.5%)	115 (36.1%)	
No	288 (71.5%)	204 (63.9%)	
Coronary Heart Disease			0.0905
Yes	88 (21.8%)	87 (27.3%)	
No	315 (78.2%)	232 (72.7%)	
COPD			0.1610
Yes	43 (10.7%)	45 (14.1%)	
No	360 (89.3%)	274 (85.9%)	
WBC, ×10^9^/L	6.02 (4.33, 8.28)	7.56 (5.42, 10.75)	<0.0001
HCT, L/L	0.35 (0.30, 0.39)	0.33 (0.28, 0.38)	0.0031
RBC, ×10^12^/L	3.88 (3.26, 4.41)	3.69 (3.05, 4.27)	0.0051
Lymphocytes %	17.85 (10.90, 27.58)	8.90 (5.30, 16.80)	<0.0001
Lymphocytes, ×10^9^/L	1.01 (0.70, 1.49)	0.70 (0.44, 1.10)	<0.0001
Eosinophil Count, ×10^9^/L	0.05 (0.01, 0.14)	0.01 (0.00, 0.05)	<0.0001
Total Neutrophils, ×10^9^/L	4.15 (2.62, 6.17)	6.34 (4.05, 9.88)	<0.0001
Hemoglobin, g/L	115.0 (98.00, 131.0)	112.0 (88.00, 126.0)	0.0148
Plateletcrit, %	1.90 (1.40, 2.60)	1.90 (1.35, 2.50)	0.1718
PLT, ×10^9^/L	186.5 (132.0, 251.8)	171.0 (114.0, 239.0)	0.0142
CRP, mg/L	21.56 (6.34, 68.29)	63.80 (26.87, 131.1)	<0.0001
PCT, ng/mL	0.13 (0.06, 0.45)	0.38 (0.13, 1.83)	<0.0001
Pro-BNP, pg/mL	330.7 (122.8, 990.4)	900.5 (306.0, 2,914)	<0.0001
IL-6, pg/mL	24.80 (11.30, 61.12)	49.30 (16.59, 116.0)	0.0001
hs-cTnT, ng/mL	0.02 (0.01, 0.03)	0.03 (0.02, 0.09)	<0.0001
D-dimer, μg/mL	0.76 (0.45, 1.97)	1.83 (0.78, 5.32)	<0.0001
Albumin, g/L	36.15 (32.05, 39.80)	34.00 (30.60, 37.50)	<0.0001
ALT, U/L	18.00 (12.00, 30.00)	19.00 (12.00, 32.00)	0.5877
AST, U/L	24.00 (18.00, 37.00)	30.00 (20.00, 47.00)	0.0001
IBIL, μmol/L	4.30 (2.80, 6.50)	4.35 (2.78, 6.60)	0.9766
DBIL, μmol/L	3.90 (2.70, 5.70)	5.00 (3.20, 8.33)	<0.0001
TBIL, μmol/L	8.50 (5.90, 12.40)	9.55 (6.60, 15.43)	0.0080
Total Protein, g/L	63.65 (59.63, 67.98)	60.60 (55.50, 65.80)	<0.0001
Creatinine, μmol/L	78.00 (63.00, 108.8)	91.50 (67.00, 174.3)	0.0001
GFR, mL/min	78.89 (51.38, 94.89)	66.54 (29.93, 86.77)	<0.0001

**Note:**

COPD, chronic obstructive pulmonary disease; WBC, white blood cell; HCT, hematocrit; RBC, red blood cell, PLT, platelet count; CRP, C-reactive protein; PCT, procalcitonin; Pro-BNP, pro-brain natriuretic peptide; IL-6, interleukin-6; hs-cTnT, high-sensitivity cardiac troponin T; ALT, alanine aminotransferase; AST, aspartate transaminase; IBIL, indirect bilirubin; DBIL, direct bilirubin; TBIL, total bilirubin; GFR, glomerular filtration rate.

Compared with the mild/moderate group, the severe/critical group exhibited significantly higher levels of white blood cell count, total neutrophil count, C-reactive protein, procalcitonin (PCT), pro-brain natriuretic peptide (pro-BNP), IL-6, high-sensitivity cardiac troponin T (hs-cTnT), D-dimer, aspartate aminotransferase (AST), direct bilirubin, total bilirubin, and creatinine; but lower levels of hematocrit, red blood cells, lymphocyte percentage, lymphocyte count, eosinophil count, hemoglobin content, platelet count, albumin, total protein, and glomerular filtration rate (GFR).

### Screening of risk factors affecting severity

Univariate and multivariable logistic regression analyses evaluating the effect of variables on the severity of COVID-19 were performed. The results demonstrated significant associations between disease severity and multiple variables (Table 2), including demographic characteristics (age, respiratory rate, pulse, temperature, diastolic blood pressure), comorbidities (diabetes), and various hematological parameters (white blood cell, hematocrit, red blood cell, lymphocyte percentage, eosinophil count, neutrophil count, hemoglobin content, platelet count, C-reactive protein (CRP), Pro-BNP, D-dimer, albumin level, AST, total protein and GFR). Respiratory rate, diabetes, lymphocyte percentage, CRP, and D-dimer were independent risk factors for severe/critical forms in elderly COVID-19 patients.

**Table 2:** Logistic regression analysis of factors affecting COVID-19 severity in the elderly.

Risk factors	Univariate logistic regression			Multivariable logistic regression		
	OR	95% CI	*p*	OR	95% CI	*p*
Male *vs*. Female	1.004	[0.742–1.360]	0.978			
Age, y	1.034	[1.018–1.051]	<0.001	0.993	[0.965–1.022]	0.641
Respiration rate, breaths/min	1.149	[1.094–1.206]	<0.001	1.126	[1.038–1.221]	0.004
Pulse, beats/min	1.016	[1.007–1.025]	<0.001	1.010	[0.995–1.024]	0.184
Body Temperature, °C	1.363	[1.057–1.759]	0.017	1.130	[0.747–1.708]	0.564
Systolic Blood Pressure	0.994	[0.987–1.001]	0.114			
Diastolic Blood Pressure	0.981	[0.968–0.994]	0.003	1.006	[0.985–1.028]	0.588
Hypertension	1.322	[0.982–1.780]	0.066			
Diabetes	1.412	[1.031–1.934]	0.032	1.808	[1.044–3.130]	0.035
Coronary Heart Disease	1.342	[0.954–1.889]	0.091			
COPD	1.450	[0.923–2.277]	0.107			
WBC, ×10^9^/L	1.082	[1.045–1.122]	<0.001	1.038	[0.960–1.121]	0.350
HCT, L/L	0.974	[0.956–0.993]	0.006	1.035	[0.749–1.431]	0.8335
RBC, ×10^12^/L	0.811	[0.694–0.948]	0.009	0.975	[0.825–1.151]	0.762
Lymphocytes %	0.929	[0.913–0.945]	<0.001	0.962	[0.934–0.991]	0.010
Lymphocytes, ×10^9^/L	1.007	[0.990–1.024]	0.432			
Eosinophil Count, ×10^9^/L	0.059	[0.015–0.237]	<0.001	0.102	[0.009–1.206]	0.070
Total Neutrophils, ×10^9^/L	1.047	[1.022–1.073]	<0.001	1.014	[0.984–1.045]	0.357
Hemoglobin, g/L	0.994	[0.988–0.999]	0.024	1.032	[0.978–1.089]	0.249
Plateletcrit, %	0.869	[0.735–1.028]	0.102			
PLT, ×10^9^/L	0.998	[0.996–0.999]	0.006	0.997	[0.994–1.000]	0.086
CRP, mg/L	1.011	[1.008–1.014]	<0.001	1.006	[1.001–1.011]	0.013
PCT, ng/mL	1.008	[0.998–1.019]	0.114			
Pro-BNP, pg/mL	1.000	[1.000–1.000]	0.001	1.000	[1.000–1.000]	0.490
IL-6, pg/mL	1.001	[1.000–1.001]	0.098			
hs-cTnT, ng/mL	0.959	[0.853–1.078]	0.483			
D-dimer, μg/mL	1.127	[1.071–1.187]	<0.001	1.124	[1.042–1.213]	0.003
Albumin, g/L	0.939	[0.912–0.966]	<0.001	1.030	[0.966–1.099]	0.363
ALT, U/L	1.001	[0.998–1.005]	0.425			
AST, U/L	1.006	[1.002–1.010]	0.006	1.002	[0.997–1.008]	0.429
IBIL, μmol/L	0.996	[0.969–1.024]	0.790			
DBIL, μmol/L	1.001	[0.993–1.009]	0.863			
TBIL, μmol/L	1.000	[0.993–1.007]	0.975			
Total Protein, g/L	0.951	[0.931–0.971]	<0.001	0.983	[0.944–1.023]	0.397
Creatinine, μmol/L	1.001	[1.000–1.001]	0.190			
GFR, mL/min	0.994	[0.991–0.997]	<0.001	0.996	[0.992–1.001]	0.098

**Note:**

COPD, chronic obstructive pulmonary disease; WBC, white blood cell; HCT, hematocrit; RBC, red blood cell, PLT, platelet count; CRP, C-reactive protein; PCT, procalcitonin; Pro-BNP, pro-brain natriuretic peptide; IL-6, interleukin-6; hs-cTnT, high-sensitivity cardiac troponin T; ALT, alanine aminotransferase; AST, aspartate transaminase; IBIL, indirect bilirubin; DBIL, direct bilirubin; TBIL, total bilirubin; GFR, glomerular filtration rate.

### Establishment of prediction models

**Figure 2:**
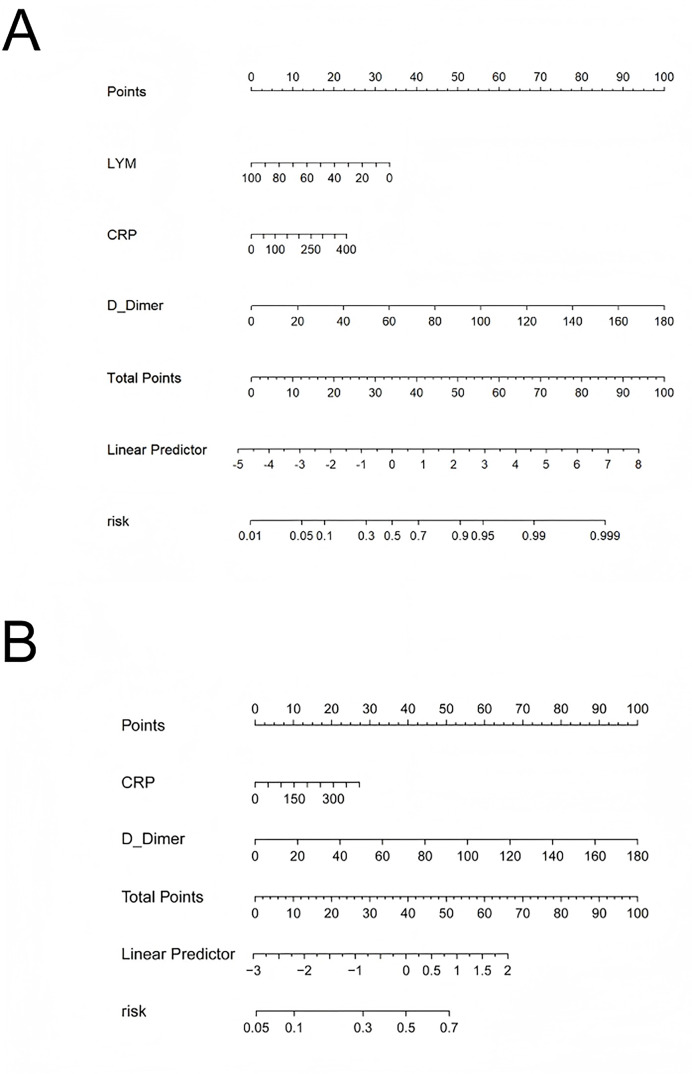
Nomograms for each outcome. (A) Nomogram for predicting severe/critical COVID-19 based on three risk factors (CRP, D-dimer and Lymphocyte Percentage). (B) Nomogram for predicting COVID-19 mortality based on two risk factors (CRP and D-dimer).

Logistic regression models were constructed using three independent severe/critical risk factors (lymphocyte percentage (LYM), CRP, D-dimer (DD)) and two mortality risk factors (CRP, D-dimer) from the laboratory examination indexes, which were visualized using nomograms (Fig. 2). Severe = −0.081 + (−0.045 * LYM) + (0.008 * CRP) + (0.074 * DD). Risk stratification followed these cut-offs: a score <0.41 indicated low risk (mild/moderate disease), while a score ≥0.41 denoted high risk (severe or critical disease).

### Model performance and validation

**Figure 3:**
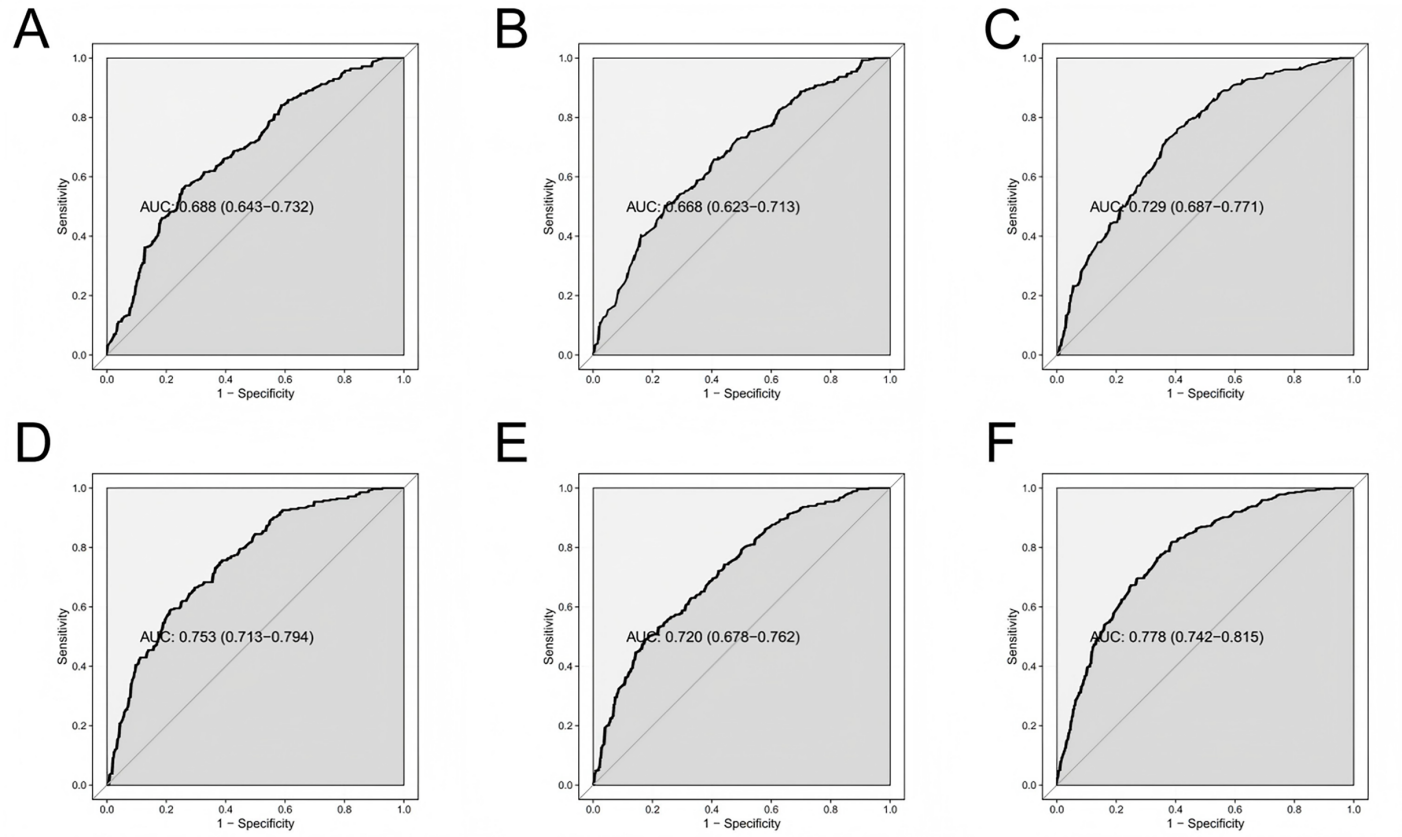
ROC curves of risk factors for predicting COVID-19 severity. (A–C) ROC analysis of a single independent risk factor based on internal test set (CRP, D-dimer, Lymphocyte Percentage). (D) ROC analysis of three independent risk factors based on internal test set (CRP, D-dimer and Lymphocyte Percentage). (E) ROC analysis of two independent risk factors based on internal test set (CRP and D-dimer). (F) ROC analysis of two independent risk factors based on external test set (CRP and D-dimer).

**Figure 4:**
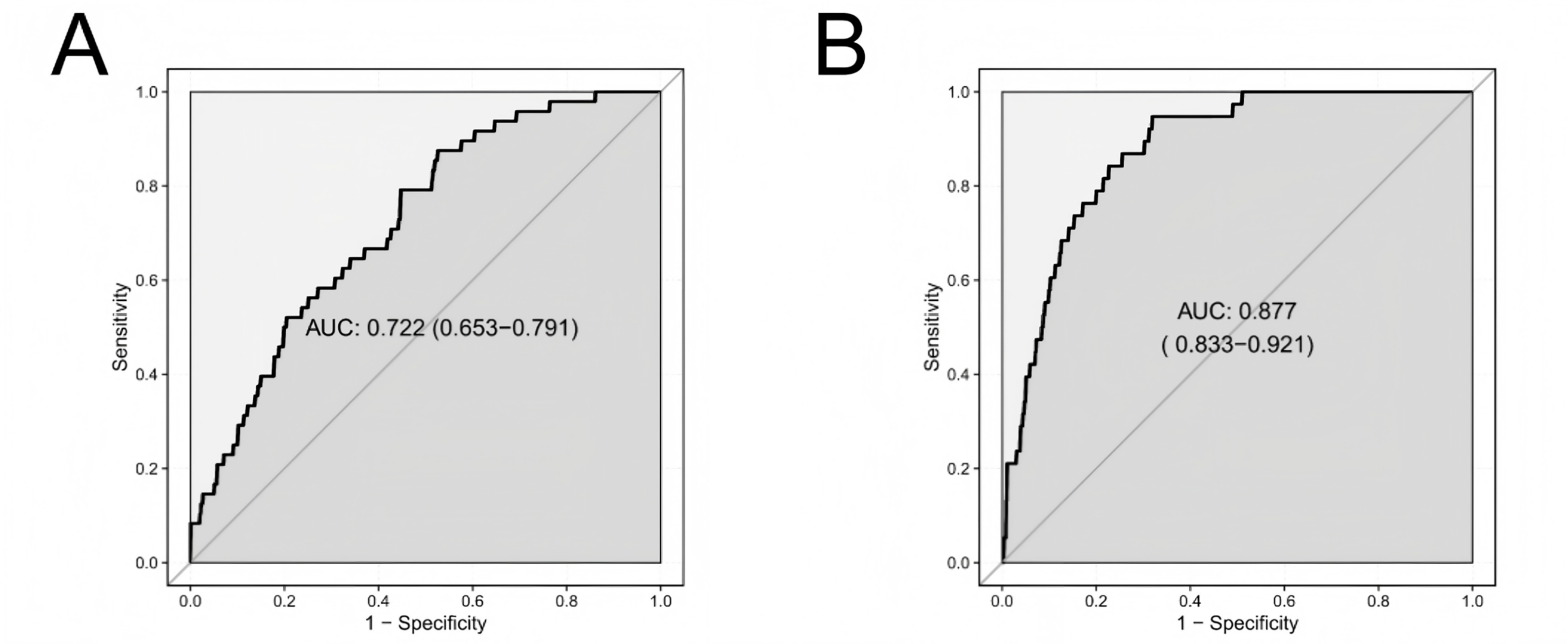
ROC curves of risk factors for predicting COVID-19 mortality. (A) ROC analysis of two independent risk factors based on internal test set (CRP and D-dimer). (B) ROC analysis of two independent risk factors based on external test set (CRP and D-dimer).

The predictive capability of the model for severe/critical COVID-19 was evaluated using ROC curve analysis. Internal validation using data from Nanfang Hospital revealed that lymphocyte percentage, CRP, and D-dimer each had significant predictive value for severe/critical outcomes in elderly COVID-19 patients (*p* < 0.05). The combination of all three markers [area under the curve (AUC) = 0.753 (0.713–0.794)] demonstrated superior accuracy in predicting severe/critical illness compared to individual indicators (Fig. 3). Additionally, the combination of CRP and D-dimer showed good predictive value for mortality in elderly COVID-19 patients [AUC = 0.722 (0.653–0.791)] (Fig. 4A). External validation using data from Huashan Hospital demonstrated that the combination of CRP and D-dimer had excellent predictive performance for severe/critical cases in elderly COVID-19 patients [AUC = 0.778 (0.742–0.815)] (Fig. 3F) and high accuracy and stability in predicting mortality [AUC = 0.877 (0.833–0.921)] (Fig. 4B).

## Discussion

Univariate and multivariable logistic analyses were performed to identify risk factors for the progression to severe/critical conditions in elderly COVID-19 patients and to construct an early prediction model, using CRP, D-dimer, and lymphocyte percentage. Given that these patients consume more healthcare resources, the model aims to optimize treatment decisions and allocate limited healthcare resources more effectively.

The elderly population is a high-risk group for severe and critical COVID-19 and a key focus of public health and preventive medicine (Shi, 2020). Simple and rapid identification of elderly patients at risk of disease progression to severe/critical forms during the initial infection phase, followed by prompt intervention, holds significant importance for reducing disease burden. Although multiple studies have developed prognostic models for COVID-19 outcomes, retrospective external validation has shown that existing COVID-19 prognosis models exhibit poor predictive performance for elderly COVID-19 patients (Zahra et al., 2024). This could be attributed to the fact that most models were trained on data covering all age groups and lacked stratified validation specifically for the elderly subgroup. Additionally, most current models are limited by their reliance on single indicators, small sample sizes, and single-source data, which restricts their broader application (Zhuang et al., 2023; Zeng et al., 2020).

Previous studies have reported independent risk factors in COVID-19 patients. For instance, a retrospective multicenter cohort study in Wuhan found that D-dimer levels greater than 1 μg/mL were associated with higher odds of in-hospital mortality, and lymphopenia was more common in severe COVID-19 cases (Zhou et al., 2020). An Egyptian study of 112 COVID-19 patients reported that hypertension and/or diabetes were associated with disease severity and adverse outcomes, and high-sensitivity C-reactive protein (HS-CRP), D-dimer, and lymphopenia were correlated with increased disease severity and mortality (Fathalla et al., 2022). A case-control study including 124 critically ill COVID-19 patients showed that lymphocyte count and CRP at admission were independent risk factors for mortality in critical COVID-19 and could serve as independent predictors of clinical prognosis (Pan et al., 2020). Another retrospective study of 274 COVID-19 patients found that non-survivors had persistent and more severe lymphopenia along with elevated inflammatory markers (high sensitivity C-reactive protein) compared to recovered patients (Chen et al., 2020). These results indicate that the three biomarkers included in our models have important clinical significance.

Consistent with previous findings, diabetes has been identified as a significant risk factor associated with COVID-19 disease severity (Charoenngam et al., 2021; Norouzi et al., 2021; Bloomgarden, 2020). A Wuhan study showed that among 52 critical COVID-19 patients, 32 non-survivors had diabetes (Yang et al., 2020). In Hong Kong, elderly type 2 diabetes mellitus (T2DM) patients (≥75 years) demonstrated higher COVID-19-related mortality than age-matched patients with cardiovascular diseases or cancer (Bloomgarden, 2020). Our study further confirmed that comorbid diabetes served as an independent risk factor for the progression to severe and critical conditions in elderly COVID-19 patients.

In a retrospective cohort study containing COVID-19 patients from 575 hospitals in 31 provinces, LASSO regression indicated that respiratory rate was a significant predictor of critical illness, while further logistic regression models demonstrated that dyspnea had significant predictive value for severe COVID-19 (Liang et al., 2020). Another study of 710 patients with SARS-CoV-2 pneumonia also found that ICU patients were more likely to experience dyspnea compared to non-ICU patients (Pan et al., 2020). Similar to the results of previous studies, this study also reported that a high respiratory rate was a relevant risk factor for adverse outcomes in elderly COVID-19 patients.

Although previous predictive models for severe COVID-19 in elderly populations have been proposed, these were predominantly derived from research cohorts with limited sample sizes (typically less than 300 patients) (Zhuang et al., 2023; Zeng et al., 2020). Our study, incorporating 1,971 patients from two medical centers, represents the largest validation cohort to date for this specific patient population. The three required variables for risk prediction (D-dimer, CRP, and lymphocyte percentage) are typically readily available upon admission. The nomograms maintained adequate calibration and discrimination ability in both the Nanfang Hospital cohort and the Huashan Hospital cohort. ROC curve analysis further confirmed the excellent predictive efficacy of the models for the risk of disease severity and death in elderly patients. Regarding the higher predictive accuracy for mortality observed in our model, this is likely because CRP and D-dimer have more direct biological associations with the underlying pathological mechanisms leading to death, such as systemic multi-organ failure and fatal thrombotic events (Ullah et al., 2020; He et al., 2021).

Severe COVID-19 represents a classic biphasic disease. The initial phase is driven by the virus, but the subsequent severe/critical phase is primarily driven by the host’s excessive and dysregulated immune inflammatory response (*e.g*., “cytokine storm”). The pathophysiological processes in this phase are largely “virus-independent” (Yang, 2025). Although viral mutations enhance immune evasion, host-specific factors (*e.g*., hypersensitive inflammatory responses, coagulation cascades) likely determine whether an individual develops severe disease (van Eijk et al., 2021). Consequently, biomarkers capturing these host pathophysiological states exhibit inherent stability across different variants. Clinical evidence supports this perspective: CRP levels show no significant difference between Omicron and Delta variants, yet elevated CRP consistently correlates with severe disease and mortality (Amini et al., 2025; Briciu et al., 2025). From the pandemic’s onset through the Omicron era, patients with elevated D-dimer persistently face higher risks of ARDS and death (Auyeung, 2025; Li et al., 2021; Varikasuvu et al., 2021). Reduced lymphocyte percentage remains highly correlated with mortality in both Delta and Omicron infections (Wei et al., 2023; Tan et al., 2020). Thus, the model based on CRP, D-dimer, and lymphocyte percentage reflects a universal host response to viral invasion rather than variant-specific characteristics. Successful validation of this model across two distinct spatiotemporal cohorts [(AUC = 0.720 (0.678–0.762), AUC = 0.778 (0.742–0.815)] further demonstrates its stability and universality. While variant evolution primarily alters transmissibility and immune evasion capabilities, it does not change the status of advanced age, comorbidities, and immune-inflammatory dysregulation as core drivers of severe disease. Consequently, this model retains significant clinical predictive value for future variants.

The developed nomogram has direct clinical utility for routine practice. It significantly assists clinicians in optimizing medical resource allocation and providing decision support for the precision management of elderly COVID-19 patients. Firstly, it enables rapid risk stratification at admission. By calculating a total score based on the patient’s CRP, D-dimer, and lymphocyte percentage, clinicians can instantly identify high-risk elderly patients, which facilitates timely escalation of care. For example, a 70-year-old patient admitted with a CRP level of 90 mg/L, D-dimer of 4.0 μg/mL, and lymphocyte percentage of 8% would receive a nomogram score of 0.575. This exceeded the high-risk threshold of 0.41, indicating a risk of severe disease. This clear signal enables the attending physician to promptly initiate intensive monitoring, consider early admission to the ICU, and prioritize aggressive anti-inflammatory or supportive therapies. Secondly, it serves as an objective aid in shared decision-making by visualizing an individual’s risk of severe outcomes. In resource-constrained settings, the nomogram offers a method to triage patients effectively, ensuring that intensive resources are allocated to those with the highest predicted need, thereby improving overall healthcare efficiency and potentially reducing mortality.

However, this study still has some limitations. First, as a retrospective study, not all patients underwent all laboratory tests. Some clinical indicators, such as cytokine levels, were not included, potentially underestimating their predictive value for disease progression and mortality in elderly COVID-19 patients. Second, it was not possible to incorporate dynamic changes in the indicators into the model for analysis. Additionally, a multi-center, large-scale research cohort is still needed to validate the results. Since the data for model development and validation entirely come from China, this may limit the generalizability of the risk score in other regions of the world.

## Conclusions

ROC curves for lymphocyte percentage, CRP, and D-dimer indicated that they were good predictors of disease severity and death. The developed and validated early prediction model for severe/critical COVID-19 in elderly patients holds significant clinical value and application prospects for enhancing precision diagnosis and treatment in this vulnerable population. However, due to the retrospective nature of the study and its limitation to a single geographic population (China), the model’s generalizability requires external validation in more diverse multinational and multiethnic elderly populations. Prospective cohort studies should be designed to directly evaluate the model’s predictive accuracy and clinical utility in forecasting severe disease among elderly patients.

## Supplemental Information

10.7717/peerj.21417/supp-1 Supplemental Information 1 Comparison of baseline characteristics between patients in the Nanfang Hospital and Huashan Hospital.

10.7717/peerj.21417/supp-2 Supplemental Information 2 Clinical characteristics of elderly COVID-19 patients in Huashan Hospital.

10.7717/peerj.21417/supp-3 Supplemental Information 3 Ordered logistic regression analysis of factors affecting COVID-19 mortality in the elderly.

10.7717/peerj.21417/supp-4 Supplemental Information 4 Raw Data.

## Additional Information and Declarations

### Data Availability

The following information was supplied regarding data availability:

The raw data is available in the Supplemental Files.
