# Supplementary material for: Development and validation of a model for early prediction of severe/critical COVID-19 in elderly patients
Source: PeerJ. 2026 Jul 9;14:e21417. doi: 10.7717/peerj.21417 (PMC13356827; doi:10.7717/peerj.21417)
Supplement: Supplemental Information 1 [file peerj-14-21417-s001.docx]

**Supplementary Tables**

**Table 1**

Comparison of baseline characteristics between patients in the Nanfang Hospital and Huashan Hospital

| Characteristic | Nanfang (n=722) | Huashan (n =1249) | *p* |
| --- | --- | --- | --- |
| Age,y | 73.00 (66.00, 81.00) | 72.00 (66.00, 81.00) | 0.781 |
| Sex |  |  | <0.001 |
| Female | 272 (37.7%) | 619(49.6%) |  |
| Male | 450 (62.3%) | 630(50.4%) |  |
| Pro-BNP, pg/ml | 599.00 (191.85, 2,287.00) | 189.00 (69.80, 918.00) | <0.001 |
| hs-cTnT, ng/ml | 0.02 (0.01, 0.06) | 0.02 (0.01, 0.04) | <0.001 |
| PCT, ml/L | 0.21 (0.08, 0.84) | 0.12 (0.05, 0.43) | <0.001 |
| Lymphocytes×10^9^/L | 0.88 (0.57, 1.31) | 1.21 (0.81, 1.66) | <0.001 |
| CRP, mg/L | 39.84 (12.52, 95.40) | 23.27 (11.15, 45.33) | <0.001 |
| D-dimer ,ug/ml | 1.19 (0.54, 3.42) | 0.71 (0.40, 1.39) | <0.001 |
| WBC, ×10^9^/L | 6.63 (4.77, 9.54) | 5.27 (3.94, 6.97) | <0.001 |
| ALT, U/L | 18.00 (12.00, 30.00) | 17.00 (12.00, 25.00) | 0.003 |
| AST , U/L | 26.00 (19.00, 44.00) | 21.00 (16.00, 28.50) | <0.001 |
| TBIL, umol/L | 9.00 (6.10, 13.90) | 8.30 (5.95, 11.90) | 0.005 |
| Creatinine, umol/L | 85.50 (65.00, 135.00) | 80.00 (63.00, 112.00) | 0.144 |
| GFR, ml/min | 72.19 (42.78, 90.70) | 75.00 (47.00, 89.00) | 0.201 |
| Continuous variable data are presented as median (interquartile ranges, IQR).  Classified variable data are presented as n(%). | | | |
| Wilcoxon rank sum test; Pearson's Chi-squared test | | | |

Pro-BNP, pro-brain natriuretic peptide; hs-cTnT, high-sensitivity cardiac troponin T; PCT, procalcitonin;

CRP, C-reactive protein; WBC, white blood cell; ALT, alanine aminotransferase; AST, aspartate

transaminase; TBIL, total bilirubin; GFR, glomerular filtration rate
