# Supplementary material for: Development and validation of a model for early prediction of severe/critical COVID-19 in elderly patients
Source: PeerJ. 2026 Jul 9;14:e21417. doi: 10.7717/peerj.21417 (PMC13356827; doi:10.7717/peerj.21417)
Supplement: Supplemental Information 2 [file peerj-14-21417-s002.docx]

**Supplementary Tables**

**Table 2**

Clinical characteristics of elderly COVID-19 patients in Huashan Hospital

| Clinical characteristics | mild/moderate (n=922) | severe/critical (n=327) | *p* |
| --- | --- | --- | --- |
| Age, y | 70.00 (65.00, 77.00) | 79.00 (71.00, 88.00) | <0.001 |
| Sex |  |  | <0.001 |
| Female | 494 (53.6%) | 125 (38.2%) |  |
| Male | 428 (46.4%) | 202 (61.8%) |  |
| Pro-BNP, pg/ml | 131.00 (59.30, 489.00) | 657.00 (173.50, 2,566.50) | <0.001 |
| hs-cTnT, ng/ml | 0.01 (0.01, 0.03) | 0.04 (0.02, 0.10) | <0.001 |
| PCT, ml/L | 0.07 (0.04, 0.25) | 0.22 (0.08, 0.91) | <0.001 |
| Lymphocytes×10^9^/L | 1.35 (0.93, 1.77) | 0.87 (0.59, 1.25) | <0.001 |
| CRP, mg/L | 15.60 (8.43, 29.42) | 41.16 (22.14, 94.30) | <0.001 |
| D-dimer, ug/ml | 0.60 (0.36, 1.00) | 1.25 (0.60, 2.65) | <0.001 |
| WBC, ×10^9^/L | 5.08 (3.81, 6.50) | 6.04 (4.32, 8.90) | <0.001 |
| ALT, U/L | 16.00 (12.00, 23.00) | 18.00 (12.00, 31.00) | 0.002 |
| AST, U/L | 20.00 (16.00, 26.00) | 24.00 (17.00, 38.00) | <0.001 |
| TBIL, umol/L | 8.20 (6.00, 11.55) | 8.40 (5.90, 12.90） | 0.205 |
| Creatinine, umol/L | 76.00 (63.00, 99.00) | 97.00 (65.00, 200.00) | <0.001 |
| GFR, ml/min | 79.00 (57.00, 90.00) | 59.00 (23.00, 84.00) | <0.001 |

Pro-BNP, pro-brain natriuretic peptide; hs-cTnT, high-sensitivity cardiac troponin T;

PCT, procalcitonin; CRP, C-reactive protein; WBC, white blood cell; ALT,

alanine aminotransferase; AST, aspartate transaminase; TBIL, total bilirubin;

GFR, glomerular filtration rate
