# Supplementary material for: Development and validation of a model for early prediction of severe/critical COVID-19 in elderly patients
Source: PeerJ. 2026 Jul 9;14:e21417. doi: 10.7717/peerj.21417 (PMC13356827; doi:10.7717/peerj.21417)
Supplement: Supplemental Information 3 [file peerj-14-21417-s003.docx]

**Supplementary Tables**

**Table 3**

Ordered logistic regression analysis of factors affecting COVID-19 mortality in the elderly

| Risk Factors | Univariate logistic regression | | | Multivariable logistic regression | | |
| --- | --- | --- | --- | --- | --- | --- |
|  | OR | 95%CI | *p* | OR | 95%CI | *p* |
| Male vs. Female | 1.225 | 0.717-2.092 | 0.458 |  |  |  |
| Age, y | 1.069 | 1.040-1.098 | <0.001 | 1.041 | 0.996-1.089 | 0.075 |
| Respiration rate, breaths/min | 1.034 | 0.992-1.078 | 0.114 |  |  |  |
| Pulse, beats/min | 1.014 | 1.001-1.028 | 0.035 | 1.012 | 0.990-1.033 | 0.292 |
| Body Temperature, °C | 1.063 | 0.692-1.635 | 0.779 |  |  |  |
| Systolic Blood Pressure | 0.986 | 0.973-0.999 | 0.033 | 0.996 | 0.977-1.014 | 0.641 |
| Diastolic Blood Pressure | 0.988 | 0.966-1.010 | 0.278 |  |  |  |
| Hypertension | 1.503 | 0.885-2.552 | 0.131 |  |  |  |
| Diabetes | 1.342 | 0.795-2.267 | 0.271 |  |  |  |
| Coronary Heart Disease | 1.763 | 1.030-3.019 | 0.039 | 2.247 | 0.898-5.624 | 0.084 |
| COPD | 1.739 | 0.889-3.400 | 0.106 |  |  |  |
| WBC, ×10^9^/L | 1.002 | 0.993-1.011 | 0.623 |  |  |  |
| HCT, % | 0.059 | 0.003-1.344 | 0.076 |  |  |  |
| RBC,×10^12^/L | 0.801 | 0.616-1.042 | 0.098 |  |  |  |
| Lymphocytes % | 0.883 | 0.845-0.923 | <0.001 | 0.949 | 0.873-1.032 | 0.219 |
| Lymphocytes×10^9^/L | 0.567 | 0.347-0.927 | 0.024 | 0.524 | 0.127-2.162 | 0.371 |
| Eosinophil Count,×10^9^/L | 0.002 | 0.000-0.140 | 0.004 | 0.015 | 0.000-13.091 | 0.225 |
| Total Neutrophils,×10^9^/L | 1.028 | 1.013-1.043 | <0.001 | 1.042 | 1.008-1.077 | 0.016 |
| Hemoglobin, g/L | 0.992 | 0.983-1.001 | 0.085 |  |  |  |
| Plateletcrit, % | 0.732 | 0.533-1.004 | 0.053 |  |  |  |
| PLT,×10^9^/L | 0.996 | 0.993-0.999 | 0.005 | 0.994 | 0.988-1.000 | 0.040 |
| CRP, mg/L | 1.008 | 1.005-1.011 | <0.001 | 1.002 | 0.996-1.008 | 0.509 |
| PCT, ml/L | 1.003 | 0.995-1.010 | 0.478 |  |  |  |
| Pro-BNP, pg/ml | 1.000 | 1.000-1.000 | 0.044 | 1.000 | 1.000-1.000 | 0.612 |
| IL-6, pg/ml | 1.000 | 1.000-1.001 | 0.262 |  |  |  |
| hs-cTn, ng/ml | 0.967 | 0.764-1.223 | 0.777 |  |  |  |
| D-dimer, ug/ml | 1.049 | 1.025-1.074 | <0.001 | 1.040 | 1.000-1.082 | 0.049 |
| Albumin, g/L | 0.909 | 0.864-0.956 | <0.001 | 1.018 | 0.897-1.154 | 0.786 |
| ALT, U/L | 1.004 | 1.001-1.008 | 0.026 | 1.002 | 0.995-1.010 | 0.543 |
| AST, U/L | 1.004 | 1.001-1.007 | 0.009 | 1.000 | 0.996-1.005 | 0.903 |
| IBIL, umol/L | 0.982 | 0.919-1.048 | 0.581 |  |  |  |
| DBIL, umol/L | 1.004 | 0.995-1.013 | 0.359 |  |  |  |
| TBIL, umol/L | 1.003 | 0.995-1.011 | 0.494 |  |  |  |
| Total Protein, g/L | 0.945 | 0.914-0.976 | <0.001 | 1.012 | 0.933-1.098 | 0.773 |
| Creatinine, umol/L | 1.001 | 1.000-1.002 | 0.058 |  |  |  |
| GFR, ml/min | 0.982 | 0.973-0.990 | <0.001 | 0.995 | 0.976-1.015 | 0.628 |

COPD, chronic obstructive pulmonary disease; WBC, white blood cell; HCT, hematocrit; RBC, red blood cell, PLT, platelet count; CRP, C-reactive protein; PCT, procalcitonin; Pro-BNP, pro-brain natriuretic peptide; IL-6, interleukin-6; hs-cTnT, high-sensitivity cardiac troponin T; ALT, alanine aminotransferase; AST, aspartate transaminase; IBIL, indirect bilirubin; DBIL, direct bilirubin; TBIL, total bilirubin; GFR, glomerular filtration rate
